# Effects of Regular Water Replenishment on Enzyme Activities and Fungal Metabolic Function of Sheep Manure Composting on the Qinghai–Tibet Plateau

**DOI:** 10.3390/ijerph191912143

**Published:** 2022-09-25

**Authors:** Rui Cai, Xinyu Cui, Shuai Zhang, Chuncheng Xu

**Affiliations:** College of Engineering, China Agricultural University, Beijing 100083, China

**Keywords:** Qinghai–Tibet Plateau, sheep manure composting, regular water supplement, enzyme activity, fungal metabolic function

## Abstract

The dry climate characteristics of the Qinghai–Tibet Plateau will seriously affect microbial metabolism during composting. In this study, we aimed to investigate the effects of regular water supplementation on the fungal and enzymatic activities of sheep manure composting in the Qinghai–Tibet Plateau. The experiment set up the treatments of water replenishment once every 7 days(T2) and 3.5 days (T3) days, and no water supplementation was used as the control (T1). The results showed that regular water supplementation increased the activities of various enzymes during composting, and the activities of protease, cellulase, peroxidase and polyphenol oxidase in T3 were higher than those in T2. Regular water supplementation increased the relative abundance of *Remersonia* and *Mycothermus*, which were significantly positively correlated with the germination index, and degradation of organic components. Regular water supplementation could enrich fungi carbohydrate, protein, and nucleotide metabolisms, and T3 had a better effect. A redundancy analysis showed that environmental factors could significantly affect the fungal community; among them, moisture content (76.9%, *p* = 0.002) was the greatest contributor. In conclusion, regular water supplementation can improve the key enzyme activities and fungal metabolic function of sheep manure composting, and water replenishment once every 3.5 days had the best effect.

## 1. Introduction

The Qinghai–Tibet Plateau (QTP) is known as the “Asian Water Tower” and the “gene pool of biological species,” which significantly impacts the ecosystem and socioeconomic development of China and the world [1,2]. The QTP has well-developed animal husbandry, which generates large amounts of livestock and poultry waste every year. If not handled properly, this waste seriously harms the fragile ecological environment of the QTP. Aerobic composting is an effective method of converting livestock manure into high-quality organic fertilizer, reducing the toxicity and volume of the waste and allowing for the resource utilization of livestock manure [3].

However, most regions of the QTP are semi-arid and arid, and this region has the climate characteristics of dry air and rapid moisture evaporation. As moisture is an extremely important factor affecting microbial activity and the physical and chemical properties during composting [4,5], this dry climate will have a significant impact on compost production in the region. Regular water replenishment during composting is an effective method by which to solve the problem of rapid water loss. Currently, there are few reports on the impact of regular water supplementation on the composting of livestock and poultry manure in the QTP. Therefore, it is necessary to study this to enrich the knowledge in this field and provide theoretical support for the efficient utilization of livestock and poultry manure in the QTP.

Extracellular enzymes secreted by microorganisms during composting are essential active substances that reduce the volume and toxicity of the waste and facilitate resource utilization. The enzymes in compost are mainly hydrolases and oxidoreductases [6], which can catalyze the degradation of various organic matters in compost materials, promote the synthesis of humus, and reduce the toxicity of pollutants. The effective control of enzyme activity is of great significance for the utilization of organic waste resources. Characterizing the dynamics of enzymatic activities is an effective way to understand microbial degradation of organic components during composting [6], and there is extensive research being conducted on enzyme activity during composting. These studies include the effects of the carbon-nitrogen ratio (C/N) [7,8], inoculation of microorganisms [9,10], and various organic and inorganic additives [11,12,13,14] on the enzymatic activity of composting.

Fungi play an important role in the degradation of recalcitrant compounds, stabilization of organic matter, and breakdown of organic residues under dry, acidic, and low-level nitrogen conditions [15,16]. Currently, there are many studies on fungal communities in composting, including different raw compost materials [17,18,19,20], additives [21,22,23,24], and environmental factors [25,26].

To the best of our knowledge there is no study on the effect of regular water supplementation on enzyme activity and fungal metabolism during composting; therefore, this study will explore this by conducting a sheep manure composting experiment. The main purposes of the experiment were as follows: to determine (I) the effects of water replenishment on the enzyme activity (including protease, urease, cellulase, sucrase, peroxidase, polyphenol oxidase, and β-glucosidase) of sheep manure composting; (II) the effects of water replenishment on the fungal diversity and metabolic function of sheep manure compost; and (III) the relationship among fungal communities, enzyme activity, and environmental factors.

## 2. Materials and Methods

### 2.1. Composting Materials

Fresh sheep manure and rape straw were obtained from Bakatai Farm (Qinghai Province, China) and impurities such as plastic and glass were removed from the compost material. The rape straw was air-dried and crushed for use as a compost C/N ratio and porosity regulator. The key physicochemical properties of sheep manure and rape straw are summarized in Table 1.

### 2.2. Composting Design and Sampling

Sheep manure and rape straw were mixed evenly at a ratio of 4:1 dry weight (Table 1), and the initial moisture content was adjusted to 60%. Windrow composting was used in this experiment. The length, width, and height of the composting pile were 2 m, 1 m, and 0.6 m, respectively. The composting experiment lasted for 28 days (10 August to 6 September 2020) at the Bakatai Farm (at an altitude of approximately 3300 m), and all composting piles were turned once every 3.5 days. The experiment consisted of three treatments with different water supplement frequencies, which were no water replenishment (T1), water replenishment once every 7 days (T2), and water replenishment once every 3.5 days (T3). T2 and T3 were watered during the early 21 days of composting, with water replenishment of up to 60% each time. Each water replenishment of T2 and T3 was carried out simultaneously with turning to ensure an even moisture content. Multi-point samples were gathered from different depths of the composting pile on Days 1, 3, 5, 7, 10, 13, 16, 21, and 28 to improve representativeness and homogeneity. These samples were then evenly mixed and divided into two parts as follows: one was immediately stored at −20 °C to analyze enzyme activity, and the other was stored at −80 °C for DNA analysis.

### 2.3. Enzyme Activities and Fungal Community Detection

The protease activity of compost was determined using ninhydrin colorimetry [27], expressed as the number of milligrams of glycine produced after 24 h reaction with 1 g dry sample; urease activity was determined using sodium phenolate sodium hypochlorite colorimetry and expressed as milligrams of ammoniacal nitrogen (NH_4_^+^-N), formed after a 24 h reaction with 1 g dry sample [7]; and sucrase and cellulase activities were measured using the 3,5-Dinitrosalicylic acid method [27]. Sucrase activity was expressed in milligrams of glucose generated after 24 h reaction with 1 g of the dry sample, and cellulase activity was expressed in milligrams of glucose generated after 72 h reaction with 1 g of dry sample. β-glucosidase activity was determined using nitrophenol colorimetry and expressed as micrograms of p-nitrophenol formed after 1 h reaction with 1 g dry sample [28]; and the activities of peroxidase and polyphenol oxidase were determined using pyrogallol colorimetry and expressed as the milligrams of pyrogallol formed after 2 h of reaction with 1 g of the dry sample [27].

Genomic DNA was extracted from the sample (0.5 g) using a FastDNA^TM^ SPIN Kit for soil (MP Biomedicals, Irvine, CA, USA), according to the manufacturer’s instructions. The extracted genomic DNA was checked using 1% agarose gel electrophoresis. Polymerase chain reaction amplification was performed as described in a previous study [29], and the universal primer pair ITS1-F (50-CTTGGTCATTTA-GAGGAAGTAA-30) and ITS2 (50-TGCGTTCTTCATC-GATGC-30) was used to target the ITS1 region of the fungal nuclear ribosomal repeat unit. Both primer pairs contained 12-bp barcodes unique to each sample and the appropriate adapters to permit sequencing on the Illumina MiSeq platform. Library construction and the Illumina MiSeq paired-end sequencing were performed by Beijing Allwegene Technology Co. Ltd. (Beijing, China) according to the manufacturer’s instructions.

### 2.4. Statistical Analysis

Three independent replicate experiments were performed for each treatment, and SPSS software (version 23.0) (IBM, Armonk, NY, USA) was used to calculate the mean and standard deviation of the enzyme activity. Origin 2021b (OriginLab, Northampton, MA, USA) was used to analyze enzyme activities, environmental factors, and fungal associations. Figures showing each indicator were produced using Origin 2021b. The bioinformatics tool, PICRUSt2, was used to predict the function of the fungal community in the samples.

## 3. Results and Discussion

### 3.1. Changes in Physicochemical Properties

The changes of physicochemical properties during composting are based on a previous study [30]. The temperature of all treatments increased rapidly during the initial stage of composting and exceeded 50 °C on the first day (see Supplementary Materials Figure S1). The temperatures of T2 and T3 both exceeded 50 °C during the first 12 days of composting, whereas the temperature of T1 began to decrease rapidly on Day 9 due to rapid water loss. The temperature of T1 was below 20 °C after 20 days of composting and subsequently approached ambient temperature, whereas T2 and T3 were below 20 °C after 22 days and subsequently entered a thermal steady state. The temperature changes in the three treatments were closely related to the degradation of their organic components. In the early stage of composting, the organic components of the three treatments degraded rapidly. With the water loss, the degradation of the organic components of T1 tended to be stagnant, while the degradation rates of the organic components of T2 and T3 were significantly higher than those of T1 in the middle and late stages of composting.

The C/N ratio, E_4_/E_6_ ratio, and germination index (GI) are commonly used as sensitive parameters to evaluate the degree of phytotoxicity and maturity of compost. The compost maturity was negatively correlated with the C/N and E_4_/E_6_ ratios, and positively correlated with the GI. According to previous studies (see Supplementary Materials Table S1), the C/N ratio and E_4_/E_6_ ratio of T2 and T3 at the end of composting were significantly lower than that of T1, which indicated that regular water supplementation can significantly improve the maturity of compost. On Day 28, the GI of T2 and T3 were more than 120%, whereas that of T1 was less than 80%. According to the standard reported in some studies [13,31], T1 was still phytotoxic, while T2 and T3 were fully mature, and the effect of T3 was better than that of T2.

### 3.2. Changes in Enzyme Activity

**Figure 1:**
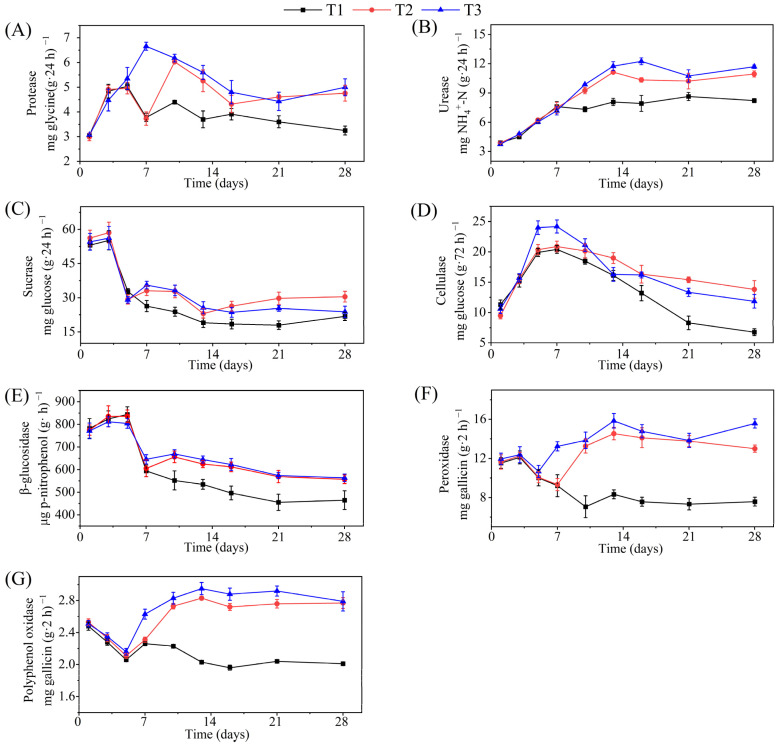
Changes in protease (**A**), urease (**B**), sucrase (**C**), cellulase (**D**), β-glucosidase (**E**), peroxidase (**F**), and polyphenol oxidase (**G**) activity during composting.

Proteases mediate the first step of mineralization, which is often the rate-limiting step in the nitrogen cycle [32]. As shown in Figure 1A, protease activity in all three treatments increased rapidly in the early stages owing to the increased availability of water-soluble nitrogen [33]. From days 5–13, the protease activity of T1 decreased rapidly, and then was maintained at a low level. The protease activities of T2 and T3 decreased after reaching the highest value; however, they were significantly higher than those of T1 at this stage. Urease is also a key functional enzyme in the nitrogen cycle that can catalyze the hydrolysis of urea to produce carbonic acid and ammonia [32,34]. Urease activity in all treatments increased rapidly during the early stages (Figure 1B). Subsequently, the urease activity of T1 tended to be stable after the 7th day of composting, while T2 and T3 increased continuously during the early 13 days and then stabilized, which led to the urease activities of T2 and T3 being significantly higher than that of T1 at the later stage of composting. The changing trend in urease activity in this study was similar to that reported in a previous study [33] both at a low level in the early stage of composting and at a high level in the late stage, indicating that urease is sensitive to high temperatures.

As shown in Figure 1C, all treatments showed high sucrase activity in the initial stage. Subsequently, the sucrase activity of T1–T3 gradually stabilized after a rapid decline. The cellulase activity (Figure 1D) of all treatments increased rapidly during the early stage and remained high during the thermophilic phase. The cellulase activity of T3 was significantly higher than that of the other treatments in the thermophilic stage, whereas T1 had the lowest cellulase activity in the middle and late stages. As shown in Figure 1E, the β-glucosidase activity of all treatments during the initial stage was high, increasing over the first five days. Subsequently, the β-glucosidase activity decreased rapidly and then gradually stabilized. Sucrase, cellulase, and β-glucosidase play essential roles in the degradation of organic components during composting [6]. Among them, sucrase can catalyze the hydrolysis of sucrose to produce glucose and fructose [10], whereas cellulase and β-glucosidase can catalyze the decomposition of cellulose into oligosaccharides or monosaccharides [9,35], which are beneficial for the decomposition and utilization of microorganisms. These three enzymes all show high activity in the early stage of composting, and catalyze the rapid degradation of organic matter in this stage. With the rapid evaporation of water in T1, the microbial activities were seriously hindered, which further affected the activities of extracellular enzymes, resulting in significantly lower enzyme activities than those in the other treatments. Regular water supplementation improved the activities of these three enzymes during composting, thus promoting the degradation of cellulose and other organic substances.

The activities of peroxidase and polyphenol oxidase in the three treatments decreased during the first five days of composting (Figure 1F,G). Subsequently, the activities of these two enzymes in T1 continued to decline, while T2 and T3 increased rapidly and remained at a high level in the later stage, and the activities of these two enzymes in T3 were higher than those in T2 after five days. It has been reported that peroxidase can oxidize the lignin polymer, while polyphenol oxidase plays an important role in the conversion of aromatic compounds, both of whose enzymes can promote humus formation [32]. Regular water supplementation can improve the activities of peroxidase and polyphenol oxidase in the compost, thereby promoting the compost maturity and the humus formation. Overall, T3 had the highest activity of these two enzymes during composting, which partly explains the optimal composting maturity of T3.

### 3.3. Fungal Diversity Analysis and Community Structure

**Figure 2:**
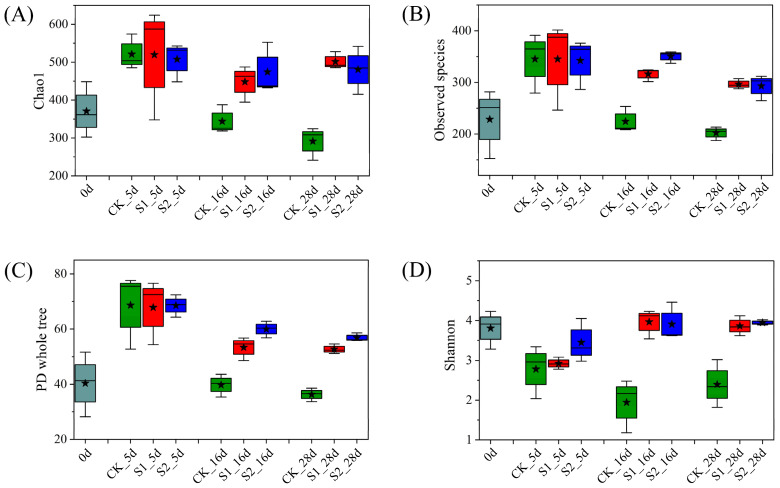
Fungal alpha diversity, (**A**) Chao1, (**B**) observed species, (**C**) PD whole tree, and (**D**) Shannon indices. ★: the average.

The Chao1 and the observed species’ indices were calculated to assess the richness, and the phylogenetic diversity (PD) whole tree and the Shannon indices were calculated to reflect the diversity [22]. As shown in Figure 2A–C, the Chao1, observed species, and PD whole tree index values of all treatments were significantly higher than those of raw materials on Day 5. With rapid water loss, the three indices of T1 decreased rapidly, whereas those of T2 and T3 remained high. The change trend of the Shannon index was different from that of other indices, and its value in all treatments showed a downward trend in the early stage of composting. Subsequently, T1 still showed a downward trend, whereas T2 and T3 showed an upward trend. The fungal alpha diversity index values of T1 were significantly lower than that of T2 and T3 in the middle and late stages of composting, suggesting that regular water supplementation could improve the richness and diversity of fungi during sheep manure composting in the QTP, which promoted composting fermentation and significantly improved the compost quality.

**Figure 3:**
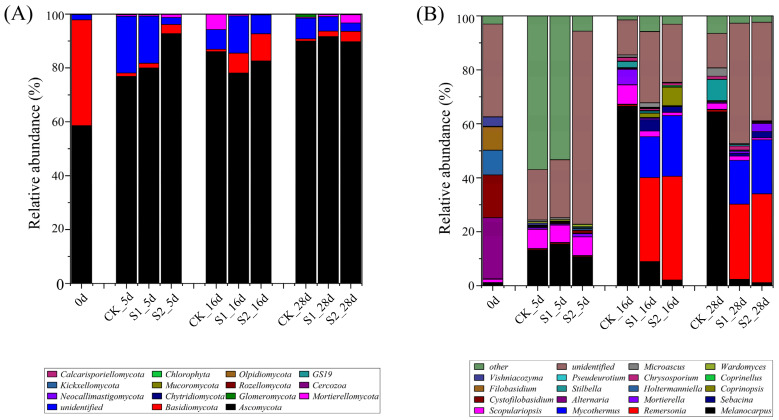
Fungal community structure at the phylum level (**A**), and at the genus level (**B**).

As shown in Figure 3A, *Ascomycota* and *Basidiomycota* were the dominant fungal phyla during composting, which is similar to the results of previous studies using other waste materials for composting [17,36,37,38]. *Ascomycota* and *Basidiomycota* have been reported to produce special stress-resistant structures that resist adverse composting environments and play an important role as decomposers during composting [23]. The relative abundance of *Ascomycota* rapidly increased during the first five days in all treatments, reaching 76.88%, 79.96%, and 92.72% in T1–T3, respectively. On Day 16 and 28, the relative abundance of *Ascomycota* was >78% in all the treatments. This indicates that *Ascomycota* continue to play a dominant role in the entire composting process. This is because *Ascomycota* has a good high-temperature adaptability and can utilize a variety of carbon sources, and is considered the dominant phylum in lignocellulosic compost ecosystems owing to the secretion of cellulase and hemicellulase [37,39,40]. Contrary to the change in the abundance of *Ascomycota*, the abundance of *Basidiomycota* decreased rapidly in the early stages of composting. Subsequently, the relative abundance of *Basidiomycota* first increased and then decreased in T2 and T3 but remained low in T1, and it seems to be more suitable for high-moisture and low-temperature environments.

Figure 3B shows the variations in the fungal community structure at the genus level. The most abundant genera (≥5%) in the raw compost materials were *Alternaria* (22.63%), *Cystofilobasidium* (15.84%), *Holtermanniella* (9.14%), and *Filobasidium* (8.62%), and their abundance declined rapidly to <2% in all the treatments during the thermophilic period. On Day 5, the relative abundance of *Melanocarpus* in T1–T3 was significantly higher than that in the raw materials. *Melanocarpus* is a fungus that can secrete cellulase, xylanase, and laccase [37,41,42], which catalyzes the rapid degradation of organic components of compost at the thermophilic stage. The abundance of *Scopulariopsis* in all the treatments peaked on Day 5, indicating that *Scopulariopsis* may play an important role in a high-temperature composting environment. On Day 16 and 28, the relative abundance of *Melanocarpus* in T1 rapidly increased to 64.49–66.64%, which was significantly higher than that in T2 and T3, while the abundance of *Remersonia* and *Mycothermus* in T2 and T3 was significantly higher than that in T1. The above phenomena indicate that regular water supplementation can increase the abundance of *Remersonia* and *Mycothermus*, which may promote the composting fermentation and the degradation of lignocellulose and other organic components in the middle and late stages.

### 3.4. Prediction of Gene Abundance Involved in Fungal Metabolic Based on PICRUSt2

**Figure 4:**
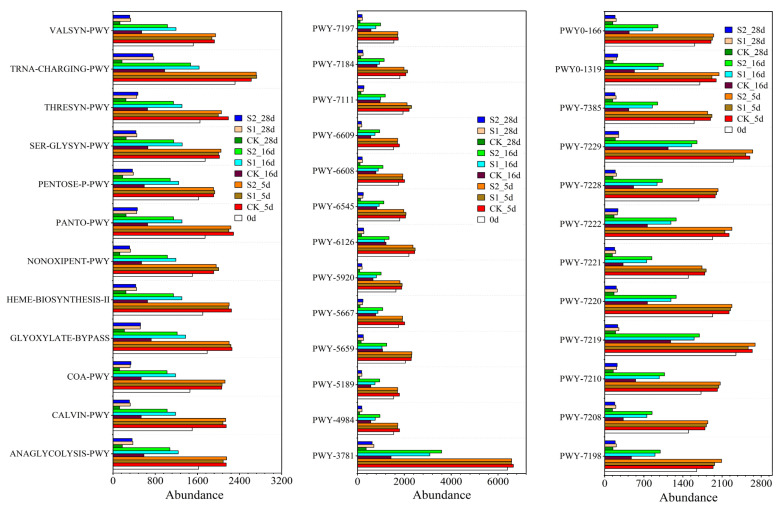
Prediction of the abundance of different metabolic pathways based on PICRUSt2.

The abundance prediction of genes related to fungal metabolic pathways is shown in Figure 4, which provides the top 37 metabolic pathways (for functional names see Supplementary Materials Table S2). The abundance of functional genes of these metabolic pathways in all treatments showed an increasing trend at the beginning of composting and then decreased rapidly and remained low at the end of composting. These results indicated that these metabolic functions of fungi had a high capacity in the early stage of composting, and then gradually decreased as the degradation of organic matter in the compost decreased. Among these pathways, aerobic respiration I (PWY-3781) had the highest gene abundance, because this pathway is an important pathway of microbial energy metabolism, which is an important process for converting energy generated by organic degradation into adenosine triphosphate (ATP). Organic component metabolism, including carbohydrate, protein and lipid metabolism, is one of the main metabolic pathways. These metabolic pathways included the glycolysis (ANAGLYCOLYSIS-PWY), pentose phosphate pathway (NONOX-IPENT-PWY, PENTOSE-P-PWY), glyoxylate cycle (GLYOXYLATE-BYPASS), urea cycle (PWY-4984), and amino acid synthesis (SER-GLYSYN-PWY, THRESYN-PWY, VALSYN-PWY). Nucleotide metabolism was also the main metabolic pathway, including PWY-6608, 6545, 7219–7222, and 7229. There was no significant difference in the abundance of these functional genes among the three treatments on Day 5; however, with the rapid decrease in water content in T1, these functional genes in T1 were significantly lower than those in other treatments. The abundance of these functional genes in T3 was slightly higher than those in T2 on Day 16, but there was no significant difference on Day 28. These results indicated that regular water supplementation can increase the abundance of genes related to major fungal metabolic pathways during sheep manure composting in the QTP, which is conducive to the fermentation and maturation of compost.

**Figure 5:**
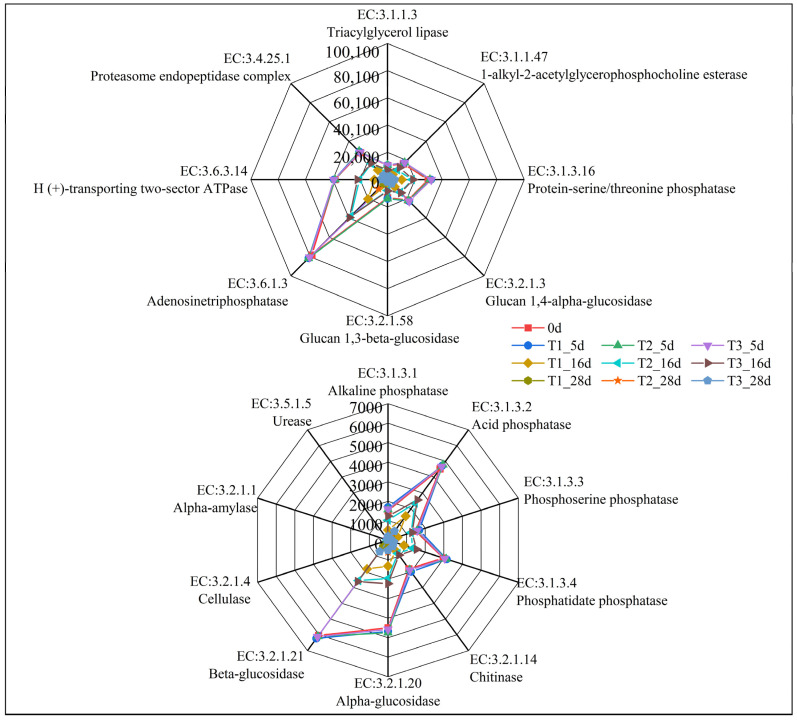
Prediction of fungal hydrolase gene abundance based on PICRUSt2.

Figure 5 shows the variation in abundance of some genes encoding hydrolases during fungal metabolism. The abundance of the genes encoding the cellulase (EC:3.2.1.4), urease (EC:3.5.1.5) and alpha-amylase (EC:3.2.1.1) of fungi was low during the entire composting process. The abundance of genes encoding other hydrolases was significantly higher than those of the first three, and they were mainly involved in protein, carbohydrate, and energy metabolism, such as protein-serine/threonine phosphatase (EC:3.1.3.16), alpha-glucosidase (EC:3.2.1.20), beta-glucosidase (EC:3.2.1.21), glucan 1,4-alpha-glucosidase (EC:3.2.1.3), glucan 1,3-beta-glucosidase (EC:3.2.1.58), and H (+)-transporting two-sector ATPase (EC:3.6.3.14), etc. The abundance of these hydrolase encoding genes also showed a similar trend during composting, with their abundance increasing at the beginning of composting and then decreasing rapidly. The abundance of these hydrolase encoding genes in T1 was significantly lower than that in the other treatments in the middle and late stage of composting, indicating that regular water supplementation may enhance the ability of fungi to degrade organic matter in sheep manure composting in the QTP.

### 3.5. Correlation Analysis between Fungus and Physicochemical Indicators

**Figure 6:**
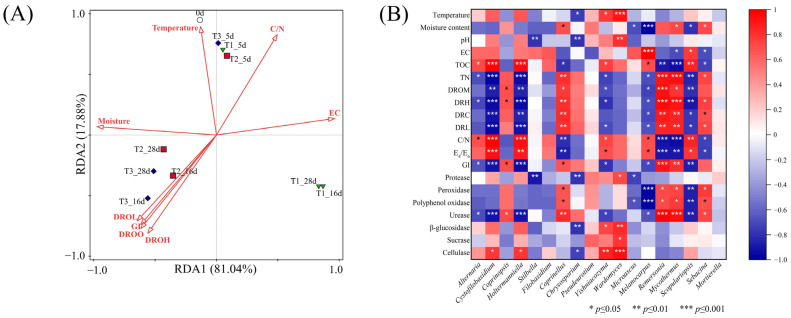
Redundancy analysis (RDA) (**A**) and correlation heat map (**B**) between fungus and physicochemical indicators. DROM: Degradation rate of organic matter; DRH: Degradation rate of hemicellulose; DRC: Degradation rate of cellulose; and DRL: Degradation rate of lignin.

The results of the redundancy analysis (RDA) between the fungus and physicochemical indices are shown in Figure 6A. The first and second axes explained 81.04% and 17.88% of the variance, respectively. All of the selected environmental factors explained 97.4% of the changes in the fungal community, indicating that environmental factors significantly influenced these changes. Among all the environmental factors, moisture content (76.9%, *p* = 0.002) was the greatest contributor, followed by the degradation rate of organic matter (17.8%, *p* = 0.002). The correlation between the remaining environmental factors (GI, C/N, and EC) and the bacterial community did not reach statistical significance (*p* > 0.05). However, this does not mean that these factors do not affect the bacterial community. Adding water during the composting of sheep manure in the QTP can improve the microbial environment, thus improving the microbial community structure in the composting pile.

Figure 6B shows the Spearman’s correlation heat map of the dominant fungus and physicochemical parameters. The abundances of *Wardomyces* and *Vishniacozyma* were significantly positively correlated with temperature, whereas that of *Chrysosporium* was significantly negatively correlated with temperature. The abundance of *Coprinellus*, *Mycothermus* and, *Sebacina* was significantly positively correlated with moisture content, whereas that of *Melanocarpus*, *Scopulariopsis*, and *Microascus* was significantly negatively correlated with moisture content. The abundances of *Coprinellus*, *Mycothermus*, and *Remersonia* were significantly positively correlated with the GI and degradation rate of organic components, and negatively correlated with the C/N and E_4_/E_6_ ratios, and total organic carbon, whereas *Cystofilobasidium*, *Holtermanniella*, *Melanocarpus*, *Vishniacozyma*, and *Scopulariopsis* had the opposite result. This indicates that these fungi are closely related to compost maturity. Regular water supplementation can change the structure of these fungi during composting, thereby improving the compost quality. Figure 6B also shows that the fungal community was significantly correlated with the enzyme activity. Protease activity was significantly positively correlated with the abundance of *Wardomyces*, and negatively correlated with that of *Stilbella*, *Chrysosporium*, and *Microascus*; peroxidase, polyphenol oxidase, and urease activities were significantly positively correlated with the abundance of *Coprinellus*, *Mycothermus*, *Sebacina*, and *Remersonia*, and negatively correlated with that of *Melanocarpus* and *Scopulariopsis*; and β-glucosidase and cellulase activities were significantly positively correlated with the abundance of *Wardomyces* and *Vishniacozyma*, and negatively correlated with that of *Chrysosporium*. These phenomena indicate that regular water supplementation can improve the fungal community structure during composting, which may further affect the secretion of multiple functional enzymes, and ultimately affect composting fermentation.

## 4. Conclusions

Regular water supplementation during sheep manure composting on the Qinghai–Tibet Plateau can improve the activities of various functional enzymes, increase fungal richness and diversity, and enhance the metabolic capacity of fungi. Through research, it was found that the effect of water replenishment every 3.5 days was better than that every 7 days.

## Figures and Tables

**Table 1:** Physiochemical properties of composting raw materials.

	Organic Matter Content (%)	Total Organic Carbon Content (%)	Total Nitrogen Content (%)	Carbon-Nitrogen Ratio	pH	Electrical Conductivity (mS/cm)
sheep manure	71.6 ± 0.66	39.8 ± 0.32	2.4 ± 0.02	16.6 ± 0.34	7.64 ± 0.04	2.03 ± 0.02
Rape straw	96.2 ± 0.58	53.4 ± 0.51	0.82 ± 0.01	65.1 ± 1.24	-	-
Mixture	76.5 ± 0.81	44.2 ± 0.47	2.08 ± 0.01	21.3 ± 0.86	7.52 ± 0.12	2.51 ± 0.03

## Data Availability

Data sharing not applicable.

## Supplementary Materials

The following supporting information can be downloaded at: https://www.mdpi.com/article/10.3390/ijerph191912143/s1, Figure S1: Changes in temperature during composting; Table S1: Changes in physicochemcal indicators; Table S2: Fungal top 37 metabolic pathways and their corresponding functions.
