# Supplementary material for: Effects of Regular Water Replenishment on Enzyme Activities and Fungal Metabolic Function of Sheep Manure Composting on the Qinghai–Tibet Plateau
Source: Int J Environ Res Public Health. 2022 Sep 25;19(19):12143. doi: 10.3390/ijerph191912143 (PMC9566448; doi:10.3390/ijerph191912143)
Supplement: Supplementary file 1 [file ijerph-19-12143-s001.zip › Table S1 Changes in physicochemcal indicators.pdf]

Table S1 Changes in physicochemcal indicators

Time /day	Treatment	Degradation rate of organic components/%				Moisture Content /%	pH	EC / mS·cm^{-1}	TOC /%	TN /%	$E_4/4_6$ ratio	C/N ratio	GI/%
		OM	Hemicellulose	Cellulose	Lignin								
5	T1	25.5	29.4	28.7	12.9	42.7	7.70	2.81	40.5	2.37	7.15	17.1	40.8
	T2	25.5	29.8	27.3	10.5	41.8	7.78	2.88	41.0	2.24	7.18	18.3	42.8
	T3	26.6	29.3	27.6	7.71	53.1	7.76	2.75	41.1	2.21	7.21	18.6	44.5
16	T1	32.6	35.6	33.6	14.6	26.1	7.44	3.13	39.6	2.52	6.98	15.7	68.2
	T2	40.9	42.4	43.4	23.4	53.7	7.56	2.61	38.1	2.71	6.62	14.0	104.6
	T3	44.2	44.2	47.4	24.5	54.8	7.66	2.44	37.8	2.89	6.52	13.1	114.3
28	T1	31.3	35.6	32.3	14.4	22.2	7.39	3.15	39.5	2.53	7.02	15.6	69.1
	T2	41.9	43.5	46.3	25.2	50.7	7.63	2.39	37.8	2.78	6.57	13.6	121.4
	T3	44.4	44.4	49.6	26.3	52.8	7.65	2.31	37.2	2.95	6.49	12.6	129.4

OM: Organic matter; EC: Electrical conductivity; TOC: Total organic carbon; TN: Total nitrogen; C/N ratio: Carbon-nitrogen ratio; GI: germination index.
