# Supplementary material for: Effects of Regular Water Replenishment on Enzyme Activities and Fungal Metabolic Function of Sheep Manure Composting on the Qinghai–Tibet Plateau
Source: Int J Environ Res Public Health. 2022 Sep 25;19(19):12143. doi: 10.3390/ijerph191912143 (PMC9566448; doi:10.3390/ijerph191912143)
Supplement: Supplementary file 1 [file ijerph-19-12143-s001.zip › Table S2 Fungal top 37 metabolic pathways and its corresponding functions.pdf]

Table S2 Fungal top 37 metabolic pathways and its corresponding functions.

BioCyc ID	MetaCyc Pathway	BioCyc ID	MetaCyc Pathway
ANAGLYCOLYSIS-PWY	glycolysis III (from glucose)	PWY-6545	pyrimidine deoxyribonucleotides de novo biosynthesis III
CALVIN-PWY	Calvin-Benson-Bassham cycle	PWY-6608	guanosine nucleotides degradation III
COA-PWY	coenzyme A biosynthesis I	PWY-6609	adenine and adenosine salvage III
GLYOXYLATE-BYPASS	glyoxylate cycle	PWY-7111	pyruvate fermentation to isobutanol (engineered)
HEME-BIOSYNTHESIS-II	heme b biosynthesis I (aerobic)	PWY-7184	pyrimidine deoxyribonucleotides de novo biosynthesis I
NONOXIPENT-PWY	pentose phosphate pathway (non-oxidative branch) I	PWY-7197	pyrimidine deoxyribonucleotide phosphorylation
PANTO-PWY	phosphopantothenate biosynthesis I	PWY-7198	pyrimidine deoxyribonucleotides de novo biosynthesis IV
PENTOSE-P-PWY	pentose phosphate pathway	PWY-7208	superpathway of pyrimidine nucleobases salvage
SER-GLYSYN-PWY	superpathway of L-serine and glycine biosynthesis I	PWY-7210	pyrimidine deoxyribonucleotides biosynthesis from CTP
THRESYN-PWY	superpathway of L-threonine biosynthesis	PWY-7219	adenosine ribonucleotides de novo biosynthesis
TRNA-CHARGING-PWY	tRNA charging	PWY-7220	adenosine deoxyribonucleotides de novo biosynthesis II
VALSYN-PWY	L-valine biosynthesis	PWY-7221	guanosine ribonucleotides de novo biosynthesis
PWY-3781	aerobic respiration I (cytochrome c)	PWY-7222	guanosine deoxyribonucleotides de novo biosynthesis II
PWY-4984	urea cycle	PWY-7228	superpathway of guanosine nucleotides de novo biosynthesis I
PWY-5189	tetrapyrrole biosynthesis II (from glycine)	PWY-7229	superpathway of adenosine nucleotides de novo biosynthesis I
PWY-5659	GDP-mannose biosynthesis	PWY-7385	1,3-propanediol biosynthesis (engineered)
PWY-5667	CDP-diacylglycerol biosynthesis I	PWY0-1319	CDP-diacylglycerol biosynthesis II
PWY-5920	superpathway of heme b biosynthesis from glycine	PWY0-166	superpathway of pyrimidine deoxyribonucleotides de novo biosynthesis
PWY-6126	superpathway of adenosine nucleotides de novo biosynthesis II		
